# *De novo* testicular tissue generation from non-testicular cell lines, biologic and synthetic scaffolds: Current findings and future translational applications

**DOI:** 10.3389/fcell.2022.954196

**Published:** 2022-11-02

**Authors:** Helia Hosseini, Christina DeBenedetto, Sriram V. Eleswarapu, Gladys Ng, Renea M. Sturm

**Affiliations:** ^1^ Department of Bioengineering, Los Angeles, CA, United States; ^2^ Department of Urology, Los Angeles, CA, United States; ^3^ UCLA Mattel Children's Hospital, Los Angeles, CA, United States

**Keywords:** male infertility, fertilty, tissue engineering and regenerative medicine, tissue engineering scaffold materials, disorders in sex differentiation, gender affirmation treatment, germline cells, somatic stem cells (SSCs)

## Abstract

In recent decades, reproductive science has revolutionized the options for biological parenthood for the 20–50% of infertility cases affected by male factors. However, current solutions exclude those who are infertile due to absent testicular tissue. This includes anorchic 46, XY individuals due to trauma or congenital factors and transgender men with a 46, XX genotype. There is a clinical need for methods to restore testicular function independent of pre-existing testicular tissue. This mini-review analyzes studies that have applied non-testicular cell lines to generate germline and non-germline testicular parenchymal components. While only 46, XY cell lines have been evaluated in this context to date, the potential for future application of cell lines from 46, XX individuals is also included. Additionally, the role of varied culture methods, media supplementation, and biologic and synthetic scaffolds to further support testicular parenchyma generation are critiqued. *De novo* testicular tissue generation in this manner will require a focus on both cellular and environmental aspects of tissue engineering. Put together, these studies highlight the future potential for expanded clinical, reproductive, and endocrine management options for individuals who are currently excluded from aspects of biologic reproduction most consistent with their gender identity and reproductive preferences.

## Introduction

Infertility impacts nearly 50 million couples globally. Male factor infertility is identified as the primary cause in 20–30% and as a contributing factor in 50% of total heterosexual infertile couples (Agarwal et al., 2015). Assisted reproductive techniques (ART) have revolutionized infertility care, but there are limitations. This includes methods that require the presence of germ cells from testicular tissue. Therefore, lack of functional testicular tissue in anorchic patients and transgender males excludes these individuals from reproductive techniques yielding biological children consistent with male gender identity. To address this limitation, the objective of this mini-review is to highlight areas of progress and opportunity surrounding the current state of testicular parenchymal generation using non-testicular cell lines and varied biologic or hybrid microenvironments.

## History of testicular tissue engineering

Researchers have been incrementally developing the capacity for *in vitro* spermatogenesis for more than a century. One of the earliest described methods (Champy, 1920) yielded early meiotic spermatocytes by culturing adult rabbit testis fragments in plasma. For decades, researchers preserved cell composition, microenvironment, and spatial arrangement of the testis by utilizing tissue fragments. Later in the 20th century, studies using cells from dissociated testes were published, still preserving the cell composition of testes, but aiming to replicate the microenvironment without testicular tissue. Since the 1990s, further co-culture has been evaluated by combining isolated germline cells and/or spermatogonial stem cell (SSC) with somatic cell lines. More recently, the seeding of either isolated germline cells or co-culture methods on biocompatible scaffolds has been explored to achieve *in vitro* spermatogenesis (Bhaskar et al., 2022), including further focus on the importance of not only the scaffold structure but also the culture methods and media conditions comprising the cellular microenvironment. All methods described above applied testis-derived cells to a wide array of culture conditions and have successfully yielded haploid cells. This review explores studies that in the past decade have taken these historic methods of testicular tissue engineering a step further by aiming to replicate the cell composition and/or microenvironment of the testis without the requirement of native testicular tissue or autologous testicular cell lines.

## *De novo* generation of testicular parenchymal cells

### Introduction to primary testicular cell lines

The testis serves two primary roles: reproductive and endocrine. In the reproductive context, spermatogenesis occurs in the seminiferous tubules and represents a continuum from SSC through spermatocytes, spermatids, and spermatozoa. Sertoli cells provide structural and metabolic support to the differentiating spermatogenic cells and are responsive to follicle stimulating hormone (FSH) secreted by the pituitary gland. In the endocrine context, the hypothalamic-pituitary-testicular axis includes Leydig cells and operates *via* a feedback loop comprising luteinizing hormone (LH) and testosterone to maintain androgenic homeostasis.

In this section, the most successful studies in the derivation of testicular cell lines from non-testicular progenitors and the varied microenvironments and culture conditions applied to accomplish this task are described. Study details are included in Table 1.

**TABLE 1:** Studies generating testicular parenchymal cells *de novo* using progenitors.

Article		*In vitro Methods*				*In vivo methods*			
1st Author, Pub year	Cell Line(s)	Culture Method	Culture conditions	Primary Endpoints	Key Markers, Terminal Differenttiation	Animal model	Cells and substrate	END point	Key pathologic results
Easley, 2012	Human HFF1 iPSC, H1 hESC	Co-culture: plate/2D, matrigel	SSC culture GDNF-condition media was required for differentiation	7–15 Days	VASA and DAZL+germ cells, subset progressed to post-meiotic (haploid), acrosin-positive differentiation	-	-	-	-
Cai, 2013	Mouse iPSC-4.1, mESCH1.2	1. Embryoid body formation, hanging drop method 2. 2D culture with MEF feeder layer	miPSC/ESC culture medium wirh FGF, RA, SCF	1.5–10 Days 1.Up to 3 months	Germline competence established of cell line (Oct4, C-kit, MVH+), PGC-like status with AP activity	Donor ICR neonatal Mice (testis), Recipient balb/c-nu/nu mice, dorsal site	Xenograft neonatal testicular tissue extract with iPSC Matrigel suspension	2 to 10 weeks	Seminiferous tubule structures containing spermatogenic cells, verified miPSC-4.1 origin of MVH+ cells
Yang, 2012	Mouse Tg-EGFP-iPSC-11.1	Embryoid body formation, hanging drop method	Importance of RA demonstrated For grem cell differentiation	5 days Without LIF, 5 days With vs without RA	PGCs were derived at greatest concentration from ra-treated eb, terminal differentiation:spermatocyctes (VASA, cKIT, SCP3+)	Icr nude mice (balb/c-nu/nu), dorsal site	Xenograft neonatal testicular tissue extract with iPSC Matrigel suspension	4 to 12 weeks	Seminiferous tubules containing germ cells (VASA, SCP3+; 12-week with spermatocytes)
Ishikura, 2021	Epiblast-like cells (EpiLC) From mESC	1. EpiLC from mESC, 2D culture 2. m PGCLs induced from EpiLC, Floting condition 3. mPGCLs with fetal testes into seminiferous tubules of W/W^v^ mice	1. EpiLC medium with fibronectin 2. mPGCLC medium, MEF layer 4. Fragemented testes, agarose gel	1) n/a 2) 4–6 days 3) 7 days 4) 8 weeks	Proceeded from PGC differentiation into gonocytes (epigenetic reprogramming) to spermatogonium/GSCs to spermatogenesis; haploid round spermatids contributes to fertile offspring vai ICSI	Donor fetal testes, recipient adult mice without endogenous spermatogenestsis, site	GSCLC suspension in GSC/GSCLC medium	8 to 10 weeks	GSCLCs proceeded to spermatogenesis following transplantation into testes
Robinson, 2021	Human iPSCs	1. 2D culture for individual cell line differentiation 2. Co-culture plating overnigt, followed by suspention organoin culture	1. Derived Sertoli, Leydig, endothelial, pertubilar myoid, and SSCs, cell-specific methods/media 2. StemPro-34 (FSH, LH, testostrone, BMP4, SCF, RA, EGF, LIF)	12 days (organoid culture)	Derived testicular cell lines self-organized into tubular structures with post-meiotic sperm atids and mature leydig and sertoli cell in organoid culture	-	-	-	-
Anand, 2014	Mouse VSEL	-	-	-	-	Busulphan-treated Swiss mice (native VSELs persisted)	Syngeneic sertoli Cells and bone Marrow derived MSCs, intratubular	2 months	Restored spermatogenesis and neotubule formation occurred only in testes with transplantaiton of Sertoli/MSCs, sperm with motility and attachment to oocytes in bencthop IVF
Shlush, 2017	Human Umbilical cord FTM and PVCs	Expanded in 2D culture in 5-step diffferentiation process; steps 4–5 co-culture with Sertoli and epididymal cell; Collagen I/IV substrate	Key supplements by steo: 1. RA 2. LIF, GDNF 3. Putrescine 4–5. Testostrone, FSH	5 weeks total differentation	Round spermatid cells (DAZL, VASA, PRM1, ACR and ODF2+) and sertoli-like cells (FSHR+); undifferentiated HUCPVCs secreted key factors regulating spermatogenesis	Busulphan-treated NOD/SCID mice	Step 3 ftm HUCPVCs (labeled), injected rete testis	6 weeks	Intratubular cells DAZL+ near basement membrane, other cells localized to interstitial spaces
Dissanayake, 2018	Human Umbilical cord MSC	2D culture	DMEM, FBS, L-glutamine, steptmycin, penicillin, Sertoli cell conditioned medium, adult	5 weeks	Str8, scp3, acr, prm1 and acrosome-like structures, elongating spermatids	-	-	-	-
Cakici, 2013	Rat adipose MSC	-	-	-	-	Busulphan-treated male wistar rats	Gfp+rat adipose derived msc suspension, unilateral rete testis injection	12 weeks	Full recovery of spermatogenesis, and continious generations of offspring were obtained via mating
Hou, 2015	Human Bone Marrow MSC	2D culture	HMG/LH.HCG, PDGF, IL-1α conditioned media	8–13 days	Leydig cell differentiation with increased markers noted vs control in vitro, 3β-hsd expression and testosterone excretion	-	-	-	-

### Induced pluripotent and embryonic stem cells (iPSC, ESC)

iPSC cell lines are derived from adult tissues that, after genetic manipulation with a core set of genes, can be expanded and differentiated into organ-specific cell lines (Doss and Sachinidis, 2019). Given their versatility, iPSCs are one of the most common cell lines applied to date for testicular parenchymal tissue generation from progenitor cell lines.

Culturing iPSCs has demonstrated the importance of culture method and conditions to generate both germ cell and non-germ cell lines. In 2-dimensional (2D) co-culture conditions, human iPSC and ESC cell lines were cultured in SSC conditions with GDNF (Glial cell line-derived Neurotrophic Factor). Differentiation was demonstrated by presence of germ cell markers followed by a subset differentiated to round spermatids (Easley IV et al., 2012). Small animal model cell lines have likewise demonstrated iPSC differentiation potential. In another study, cell lines were extracted from embryoid bodies cultured by hanging drop method and derived from iPSCs and mouse ESCs. Subsequently, an iPSC-matrigel suspension was dorsally injected in immunodeficient mice. The resultant grafts displayed ectopic seminiferous tubule formation and differentiation to post-meiotic germ cells (Cai et al., 2013). In a similar study initiating culture *via* embryoid bodies followed by transplantation of cells with testicular tissue into host mice, seminiferous tubule-like structures were likewise observed (Yang et al., 2012).

Two more recent iPSC applications have advanced further toward the ultimate objective of deriving an effective testicular environment. In one of these studies, various testicular cell types including spermatogonia, Leydig, peritubular myoid, and endothelial cells were derived from human iPSCs using cell-line specific 2D culture media conditions. Resulting cells were then co-cultured as organoids, leading to formation of tubular structures, mature somatic cell, and post-meiotic gametes (Robinson et al., 2021). Ultimately the promise of restored fertility has also occurred with the application of iPSCs in an animal model. In this study, mouse PGCLCs (Primordial Germ Cell-like Cells) were differentiated, expanded, and co-cultured with reconstituted testes (single cell layer of neonatal somatic testicular cells), followed by transplantation of derived Gonadal Stem Cell-like Cells (GSCLCs) into the testes of male mice lacking endogenous spermatogenesis. This subsequently resulted in haploid round spermatids and led to viable offspring using intracytoplasmic sperm injection (ICSI) (Ishikura et al., 2021).

The above-mentioned studies affirm the differentiation potential of iPSCs to generate haploid gametes as well as somatic cells. Future studies that confirm the genetic stability, safety of implantation, and efficacy of gametes in producing progeny are needed to improve understanding of iPSC translational potential.

### Very small embryonic-like stem cells (VSEL)

Another potential cell line for clinical translation are VSELs, a relatively rare cell population in the gonads. VSELs are regarded as a pluripotent and quiescent (during steady state) subpopulation among SSCs, comprising ∼0.03% of testicular cells and sharing numerous PGCLC markers (Ratajczak et al., 2019). In addition to the testes, VSELs can be found in bone marrow and other adult tissues, entering the cell cycle during times of stress (Kassmer and Krause, 2013).

After induction of azoospermia in mice *via* injection of the alkylating agent busulphan, the VSEL population persists within the testes. However, VSELs did not spontaneously differentiate until allogeneic Sertoli cells were transplanted, suggesting that paracrine signaling between Sertoli cells and the VSEL population may be key for resumption of spermatogenesis. These findings emphasize that supporting cell lines are critical components of a microenvironment in which VSELs can differentiate into germ cell lines capable of in vitro fertilization (IVF) (Anand et al., 2014). VSELs are a potentially valuable autologous source of progenitor cells for spermatogenesis resumption requiring further evaluation.

### Mesenchymal stem/progenitor cells (MSC)

MSCs derived from multiple sources have generated germline and non-germline testicular cells, supporting reproductive and hormonal function while providing microenvironment mediation. Several studies have demonstrated the potential for targeted MSC differentiation to germ cell lines; like iPSCs the importance of the culture method and conditions cannot be over-emphasized. However, unlike iPSCs most studies noted the use of 2D culture methods. Umbilical cord derived MSCs have been differentiated using varied 2D culture methods. *In vitro*, cell differentiation to small round cell morphology expressing a range of pre, meitoic, and post-meiotic markers has been observed. When injected into the rete testis of azoospermic mice, transplanted cells migrated to intratubular spaces and differentiated to germline cells. (Shlush et al., 2017; Dissanayake et al., 2018). Adipose derived MSCs have also demonstrated germline differentiation when injected into the rete testis of rats with azoospermia. Particularly promising for future translation, restoration of male rat fertility was demonstrated by production of live offspring (Cakici et al., 2013).

Additional progress has been made in deriving non-germline cell types from MSCs. In a study using umbilical cord derived MSCs to produce male gametes, cells expressing Sertoli-specific markers were observed during *in vitro* differentiation (Shlush et al., 2017). Additionally, *in vitro* culture of bone marrow derived MSCs in a cell-specific media resulted in significant expression of 3β-hydroxysteroid, a Leydig cell specific antigen, in differentiated cells (Hou et al., 2016).

Taken together, these studies establish the feasibility of differentiating MSCs into a range of testicular cell types, with the potential to develop both reproductive and hormonal function in infertile animal models. However, only a single study restored fertility using these methods. Recapitulating the testicular microenvironment is critical in establishing the reproductive potential of MSCs for individuals lacking testicular tissue.

### Generation of testicular cell lines from 46, XX individuals

In the process of fetal development, primordial gonads were classically described as bi-potential (Adams and McLaren, 2002). In the determination process of gonadal differentiation, the SRY (Sex Determining Region Y) gene plays a major role in testicular development. In fact, manipulating genes within the SRY pathway can result in 46, XX primordial gonads that develop a testis-like structure (Ottolenghi et al., 2007). A more recent study examined whether testicular tissue development and spermatogenesis could be induced in mice without the presence of a Y chromosome. Transgenic modifications of SRY, SOX9 (*SRY-Box Transcription Factor*), and Eif2s3x (eukaryotic initiation factor), homologs for SRY on the X chromosome resulted in mice with evidence of spermatogenesis despite absence of the Y chromosome. One of the modifications yielded spermatids that enabled production of live offspring (Yamauchi et al., 2016), indicating the potential of genetic modifications to change the fate of PGCs from 46, XX individuals.

While there have been no such studies using human tissues, investigation of genetic mechanisms in individuals with conditions resulting in differences in sexual development likewise indicate the importance of SRY and downstream effector genes on testicular parenchyma development (Wein, et al., 2012). SOX10 overexpression (Polanco et al., 2010) and R-spondin (Rspo1) frameshift mutation (Parma et al., 2006) are examples of such genes observed as drivers of male phenotype in 46, XX individuals.

## Recreating the testicular microenvironment: Biologic and structural factors

### Introduction to the use of scaffolds in testicular tissue engineering using non-testicular cell lines

A potential regenerative option for those lacking functional native spermatogenesis is to populate a 3-dimensional (3D) construct consisting of either a biologic or synthetic scaffold with select cell lines. In the first section the outcomes of recellularization of biologic scaffolds with stem cells will be discussed; the subsequent section will discuss advancements in synthetic scaffold engineering. Study details are included in Table 2.

**TABLE 2:** Studies evaluating methods to recreate the testicular microenvironment.

Article		*In vitro methods*				*In vivo methods*			
1st Author,Pub year	Cell Line(s)	Culture Conditions	Media additives	Primary Endpoints	Key Markers, Terminal Differentiation	Animal Model	Cells and Substrate	End Point	Key Pathologic Results
Movassagh, 2020	Human SSC (brain death donors, testes)	1) 2D gelatin coated dishes 2) 3D decellularized sheep testis matrix (1%SDS)	1) Media with GDNF, EGF, LIF, bFGF 2) Media with insulintransferrin-selenium, RA, Vit C/E, pyruvate, LH, FSH, testosterone	1)4 weeks 2)4–6 weeks	Significantly higher SSC proliferation and differentiation to pre-meiotic (OCT4, PLZF), meiotic (SCP3, Boule), and post-meiotic (Crem, Prot2) in 3D vs 2D cohort	-	-	-	-
Vermeulen, 2018	Human Sertoli cell	1) 2D alone 2) indirect (2D) and 3) direct seeding (3D) of decellularized porcine prepubertal testicular matrix (SDS, Triton and Trypsin, and Trypsin, varied concentrations and combinations)	Media with HEPES, Lglutamine, FBS	Day 1 to 18 endpoints	Sertoli cells demonstrated greatest proliferation at d1 and SCF secretion at d18 with vs without presence of scaffold, round-shaped structures at d18 in certain specimens may represent seminiferous cord-like structure formation	-	-	-	-
Kargar- Abarghouei, 2018	Rat bone marrow MSC	1) 2D monolayer 2) 3D decellularized rat testicular matrix (1% Triton–1%SDS-1%Dnase)	DMEM stripped of FBS to prevent MSC differentiation effect	Day 1 to 14 endpoints	DAZL-negative cell proliferation in seminiferous tubules and interstitium, with site-specific ECM cell phenotypic differences (round or oval in STs vs fibroblast-like in interstitium)	Sprague-Dawley adult male rats, renal subcapsular, subcutaneous, liver, and mesenteric implant locations	MSC-seeded testis matrix	20, 40, 60 days	Donor and migrating cells remained viable in scaffolds; DAZL-positive migrated cells without differentiation to post meiotic cells
Raya- Rivera, 2008	Bovine chondrocyte	3D seeding, followed by rotating flask bioreactor; synthetic testicular shaped poly-L-lactide-coglycolide (50:50) coated polyglycolic acid scaffold	Testosterone enanthate injected in scaffold core following cartilage formation (after initial 4 weeks in bioreactor)	Seeded x5 days, 4 weeks in bioreactor, up to 40 weeks *in vitro*	Initial burst, plateau x16 weeks, then gradually declining testosterone release up to 40 weeks *in vitro*	Castrated athymic mice, scrotal implant	Chondrocytes on testicular prostheses (scaffold) loaded with testosterone enanthate	40 weeks	Physiologic testosterone levels maintained in testosterone loaded scaffold cohort up to 40 weeks
Dores and Dobrinski, 2014	Porcine neonatal testicular cell lines	-	-	-	-	Castrate nude mice, dorsal subcutaneous implant	Testicular cell suspension 1) with vs without Matrigel 2) with vs without VEGF165	24 and 40 weeks	*De novo* tubule formation observed across cohorts; increased number of tubules containing spermatogonia (proliferation) without increased neovascularization with VEGF165
Sun, 2018	Human SSC and Sertoli cell lines, men with obstructive azoospermia	2D and 3D culture methods, SSC alone vs coculture with Sertoli cells, with vs without matrigel and media containing defined factors	Defined factors added to media:knockout serum replacement (KSR), RA, SCF, testosterone	5 to 20 days	3D co-culture with defined factor addition (3D-I cohort) induced differentiation of SSCs to haploid spermatids with the capacity to fertilize oocytes, leading to development of human embryos	-	-	-	-

### The role of the extracellular matrix (ECM) as a culture substrate

As in other areas of tissue engineering, one strategy to create an optimal microenvironment has been to use testicular ECM with or without additional scaffolding, thereby maintaining its critical growth factors and additional protein components present (Siu and Cheng, 2004). When applied *in vitro*, human and porcine SSCs cultured on decellularized human ECM indeed provided a microenvironment that successfully maintained human germline progenitor cells (Murdock et al., 2019).

Not only does the ECM affect maintenance of spermatogonial cell lines; it also influences cellular differentiation and maturation. In a study comparing the culture of human SSCs on a decellularized sheep testis membrane *versus* 2D culture media, cells cultured on the biologic scaffold expressed higher levels of pre-meiotic, meiotic, and post-meiotic differentiation markers than cell lines maintained in culture media alone (Movassagh et al., 2020). In addition to growth and differentiation of SSCs, researchers have studied attachment and proliferation of Sertoli cells using decellularized testicular ECM. In this setting, Sertoli cells maintained viability on decellularized porcine testicular ECM and demonstrated attachment, proliferation, and orthotopic organization on the biologic scaffold. A decellularized animal model such as this one may be an attractive option for translational use, as it does not require human donor organs (Vermeulen et al., 2018). Further studies are needed to optimize ECM decellularization and to improve understanding of the organization and cellular behavior of each component of the healthy testis in an *in vitro* environment.

To demonstrate biocompatibility and feasibility of biologic scaffolds for *in vivo* implantation, decellularized rat testicular ECM was cultured with bone marrow derived MSCs followed by transplantation to several different anatomic locations within the rat (renal subcapsular, subcutaneous tissue, liver, and mesentery). Constructs implanted into liver and mesentery remained intact with demonstrated biocompatibility. Additionally, vascularized constructs contained multiple classes of non-germline testicular cells. This supports the potential to use *in vivo* models as functional bioreactors to achieve cellular differentiation of non-germline testicular cells (Kargar-Abarghouei et al., 2018).

### *De novo* synthetic scaffolds in generation of testicular parenchymal components

While decellularized tissue matrices can provide a microenvironment conducive to differentiation and propagation of testicular germline and non-germline cells, future clinical implementation of decellularized tissue is limited by the challenge of scaling this technology for high volume tissue production. An alternative approach is to develop and apply *de novo* synthetic, polymer-based biomaterial scaffolds to support select cell lines. The inherent reproducibility and scalability make synthetic scaffold technology an attractive alternative.

An example of a hybrid biomaterial synthesized scaffold developed to support testosterone delivery was created by seeding a poly-l-lactic acid (PLLA) coated polyglycolic acid (PGA) polymer with chondrocytes to support cartilage development. Following an initial culture period, the scaffold was loaded with testosterone enanthate. The scaffold sustained hormonal elution *in vitro* and *in vivo,* thereby demonstrating the potential to apply scaffolds to retain physiologic levels of intratesticular testosterone. Such hormonal-structural microenvironments will be critical to increase functionality and efficiency of spermatogenesis. (Raya-Rivera et al., 2008). Another study aimed to investigate the role of a growth factor during testicular tissue formation and spermatogenesis in a synthetic scaffold. Varied concentrations of germline cells were seeded onto a Matrigel scaffold with and without added vascular endothelial growth factor (VEGF-165), followed by subsequent implantation into castrate immunocompromised mice. Due to increased tubules with spermatogonia in growth factor containing constructs, this experiment led the authors to conclude that VEGF may have a protective role against transient hypoxia during testicular tissue formation and spermatogenesis (Dores and Dobrinski, 2014).

Further studies using Matrigel-based scaffolds indicated that these findings are not isolated to animal cell lines. Human SSCs differentiated into spermatids when cultured on a 3D scaffold. These spermatids then fertilized isolated mouse oocytes *via* ICSI (Sun et al., 2018). Taken together, synthetic scaffolds can significantly enhance the function of hybrid constructs and may play a role in future restoration of both endocrine and reproductive function for individuals lacking native functional testicular tissue.

## Discussion

*In vitro* spermatogenesis has been a topic of growing interest in the field of stem cell biology. As in many areas of tissue engineering, success in early studies of induced spermatogenesis have been defined by their recapitulation of key components of the structure and biology of the testicular microenvironment. However, re-creating the *in vivo* milieu using synthetic approaches is a considerable challenge. The research studies evaluated in this mini-review focused on progress toward establishment of the testicular niche, resulting in differentiated germline and non-germline testicular cell lines derived from non-testicular cells. The ultimate goal of this work is to provide a new option for individuals who desire fertility consistent with a male gender identity without the requirement of testicular tissue.

The studies presented vary in key components that can inform future directions in testicular tissue engineering. One is their use of 46, XY progenitor cell line selection. iPSCs have been extensively studied and demonstrate particular promise, and this is anticipated regardless of 46, XX versus 46, XY source. Varied sources may however have divergent differentiation potential and this does require further evaluation. VSELs have high differentiation potential but are limited by relative scarcity and uncertainty regarding their reproductive potential. MSCs are an attractive option due to their ease of isolation and ubiquitous presence in adults. However, further evaluation of relative cell line source does require evaluation. Although future studies are needed, MSCs may be a feasible and optimal translational candidate, particularly as genetic alterations required by iPSCs may not be required in these cell lines. Future studies are needed to evaluate the use of 46, XX cell lines and the genetic alterations that may be required to generate each component of testicular parenchyma in an autologous manner using these sources.

Another critical aspect by which these studies varied was in cell and tissue culture practices, including their structural and biologic environments. Key areas of variation included the use of 2D or 3D culture, culture methods (e.g air-liquid interface, drop culture, organoids), media selection and steps in its use, and the role that cell-cell interactions and the secretome of each cell in culture may have had in co-culture studies. Additionally, the epigenome of cells impacts the yield of their differentiation to higher levels and using pharmaco-epigenetic agents, the differentiation yield can be significantly influenced (Ishikura et al., 2021). Finally, it is important that the substrate and/or scaffold on which cells are differentiated recapitulates key components and structure of ECM, with multiple aspects requiring future research into their independent and combined effects (Smith and Ma, 2004).

**FIGURE 1:**
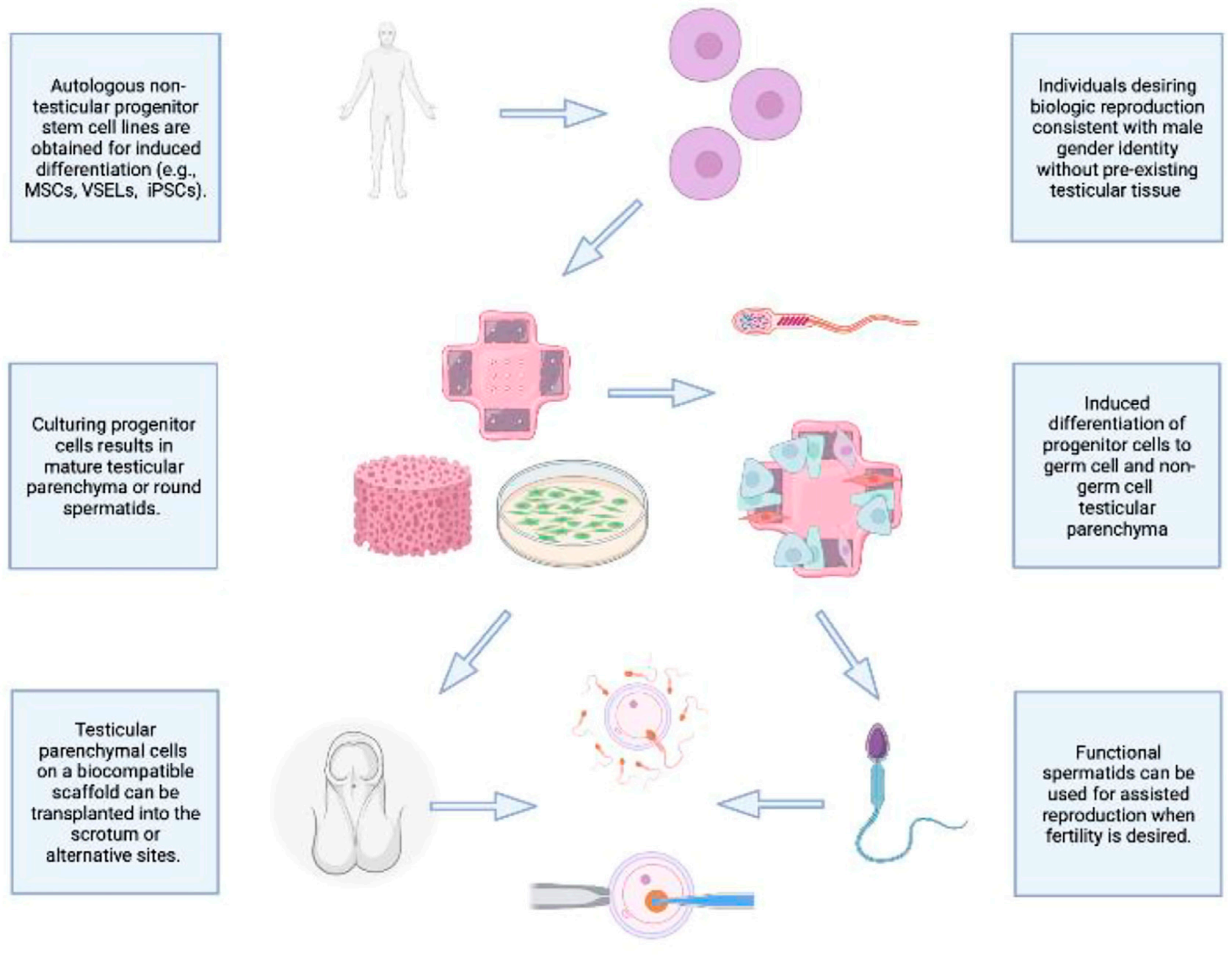
Envisioning the future of *de Novo* testicular tissue generation.

As demonstrated in Figure 1, an ideal future prospect would be to combine the best performing cell line and scaffold-based culture in a manner that supports prolonged biologic function following cellular differentiation. The priority will be to achieve reproducible, efficient *in vitro* generation of fully differentiated haploid gametes from a non-testicular progenitor cell line derived from 46, XX or 46, XY individuals. To achieve this, seeding in a 3D environment using a scaffold that emulates key components of the healthy testis microenvironment and structure will be invaluable, including promotion of cell-cell interactions. The resulting structure can be adapted for auto-transplantation, thereby supporting gametogenesis and endocrine functions of a healthy testis in an autologous, biocompatible manner. While this prospect is currently out of reach, several key independent functions of the ultimate structure have been demonstrated, including maintenance of testosterone physiological concentration, cell differentiation of progenitor cells into both germline and non-germline cells using a biological scaffold, and testis-like structural support generation for progenitor cells transplanted into host animals. As this work proceeds, it will be key to evaluate each step for its potential short- and long-term effects on epigenetic and genetic stability and transmission for effective and safe biologic reproduction for all individuals, regardless of gonadal presence or gender identity.

## Abbreviations

BMP4, Bone Morphogenic Protein 4; c-MYC, c-Myelocytomatosis Oncogene; CREM, cAMP Responsive Element Modulate; DAZL, Deleted in Azoospermia-Like; DMEM, Dulbecco’s Modified Eagle Medium; EB, Embryoid Body; ECM, Extracellular Matrix; EGF, Epidermal Growth Factor; EGFP, E-green Fluorescent Protein; Eif, Eukaryotic Initiation Factor; ESC, Embryonic Stem Cell; FBS, Fetal Bovine Serum; FGF, Fibroblast Growth Factor; FSH, Follicle Stimulating Hormone; FTM, First Trimester; GDNF, Glial-Derived Neurotrophic Factor; GFP, Green Fluorescent Protein; GSC, Gonadal Stem Cell; GSCLC, Gonadal Stem Cell-like Cells; HCG, Human Chorionic Gonadotropin; HEPES, 4-(2-Hydroxyethyl)-1- Piperazineethanesulfonic Acid; HFF, Human Foreskin Fibroblasts; HMG, Human Menopausal Gonadotropin; HSD, Hydroxysteroid Dehydrogenase; ICR, Institute of *Cancer* Research; ICSI, Intracytoplasmic Sperm Injection; iPSC, Induced Pluripotent Stem Cells; IVF, In vitro fertilization; KLF, Kruppel-like Factor; LH, Luteinizing Hormone; LIF, Leukemia Inhibitory Factor; MEF, Mouse Embryonic Fibroblasts; MSC, Mesenchymal Stem/Progenitor Cells, MVH: Mouse VASA Homologue; OCT-4, Octamer Binding Protein 4; PDGF, Platelet Derived Growth Factor; PGA, Polyglycolic Acid; PGC, Primordial Germ Cell; PGCLC, Primordial Germ Cell-like Cells; PLLA, Poly-l-lactic Acid; PLZF, Promyelocytic Leukemia Zinc Finger; PRM1, Protamine 1; PSC, Pluripotent Stem Cells; PVC, Perivascular Cells; RA, Retinoic Acid; Rspo1, R-spondin; SCF, Stem Cell Factor; SCP3, Single Cell Protein 3; SDS, Sodium Dodecil Sulfate; SFM, Serum Free Media; SOX, Sry-related HMG box; SRY, Sex Determining Region Y; SSC, Spermatogonial Stem Cells; VEGF, Vascular Endothelial Growth Factor; VSEL, Very Small Embryonic-Like Stem Cells.
